# Biogenic synthesized CuO nanoparticles and 5-fluorouracil loaded anticancer gel for HeLa cervical cancer cells

**DOI:** 10.1186/s11671-024-04166-7

**Published:** 2024-12-27

**Authors:** Gouranga Dutta, Santhosh Kumar Chinnaiyan, Thirunavukkarasu Palaniyandi, Abimanyu Sugumaran, Damodharan Narayanasamy

**Affiliations:** 1 Department of Pharmaceutics, SRM College of Pharmacy, SRM Institute of Science and Technology, Kattankulathur, Tamil Nadu 603203 India; 2 Department of Pharmaceutics, Rajiv Gandhi Institute of Pharmaceutical Sciences and Research (RPISAR), Trikaripur, Kasargod, Kerala 671310 India; 3 Department of Biotechnology, Dr. M.G.R Educational and Research Institute, Chennai, Tamil Nadu 600095 India; 4 Department of Pharmaceutical Sciences, Assam University, Silchar, Assam 788011 India

**Keywords:** CuO NPs, Cervical cancer, 5-fluorouracil, Hela cells, Pectin, Vaginal gel

## Abstract

Cervical cancer remains a significant health challenge in developing countries are high due to low HPV vaccination rates, delayed diagnosis, and restricted healthcare access. Metal nanomaterials, such as copper oxide (CuO) nanoparticles (NPs), have shown significant promise in cancer therapy due to their ability to induce apoptosis. 5-Fluorouracil (5-Fu) enhances the cytotoxic effect against cervical cancer, working synergistically with CuO NPs to maximize the therapeutic impact while potentially reducing the 5-Fu's systemic side effects. This study explores the synergistic therapeutic potential of green-synthesized CuO NPs combined with 5-Fu in a gel formulation for targeted anticancer activity against HeLa cervical cancer cells. CuO NPs were synthesized using *Trichosanthes dioica* dried seeds extract and incorporated into a pectin-xanthan gum-based gel. The green-synthesized CuO NPs exhibited a zeta potential of −23.7 mV, a particle size of approximately 26 nm, and spherical morphology. Characterization studies, including FTIR, viscosity, spreadability, pH, and stability assessments, confirmed the gel's suitability for vaginal delivery. *In-vitro* drug release showed xanthan gum extended the release up to 8 h. The MTT assay revealed PXFCu6 gel's IC_50_ at 11.82 ± 0.22 μg/mL, significantly more cytotoxic to HeLa cells, being 3.62 times potent than CuO NPs (IC_50_: 42.8 ± 0.24 μg/mL) and 1.63 times potent than 5-Fu alone (IC_50_: 19.3 ± 0.49 μg/mL). The antibacterial assay showed no inhibition for the plain gel, but *T. dioica*-mediated CuO NPs exhibited inhibition of 22.35 ± 4.9 mm. PXFCu6 gel had the more potent inhibition at 52.05 ± 1.37 mm against *Escherichia coli* growth. The PXFCu6 gel showed better stability at 4 °C, maintaining viscosity, pH, and drug release, unlike 25 °C where a mild degradation occurred. This research highlights the potential of the CuO NPs-5-Fu gel as a novel, effective therapeutic strategy for cervical cancer treatment.

**Graphical abstract:**

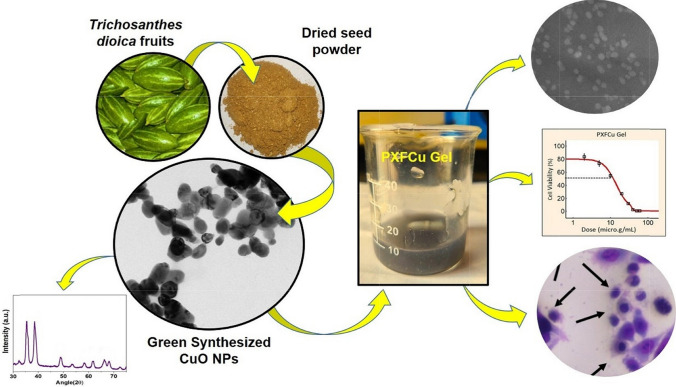

## Introduction

Cancer continues to grow globally, with rising mortality rates due to various factors. Recent studies show that in 2024, the United States alone registered over 2,000000 new cases, with 611,720 deaths [1]. Among all cancer types, cervical cancer is one of the most prevalent in women worldwide, with over 600,000 new cases reported in 2022 and 13,820 new cases in the U.S. in 2024 [1–3]. The primary cause of cervical cancer is chronic Human Papilloma Virus (HPV) infection, which affects the squamous cells in the cervix. Cervical dysplasia induced by HPV can result in malignancy if kept untreated. Some of the symptoms are aberrant vaginal bleeding, pelvic discomfort, and weight loss. The disease is prevalent in developing countries with limited access to healthcare services, examinations, and vaccines, making early detection challenging due to the asymptomatic stages [4–6]. The malignancy stage influences the treatment options available, including surgical procedures, radiation therapy, and chemotherapy. Early diagnosis of cervical cancer may lead to more favorable treatment outcomes [4, 6, 7].

Traditional cancer treatments are often unable to hinder spreading and reduce mortality. Nanomaterials have shown promise as cancer treatment agents. Drug loading and targeted distribution are more straightforward by nanoparticles with a high surface area-to-volume ratio, typically 1 to 100 nm. Metal NPs, including gold, silver, iron, zinc oxide, and copper oxide, show promise for cancer therapy, including cervical cancer [8, 9]. Pandurangan et al. studied ZnO NPs [10], and Ke et al. used gold NPs [11] on cervical cancer cell lines to demonstrate anticancer efficacy. Those nanomaterials can produce reactive oxygen species (ROS), induce apoptosis, and enhance the effectiveness of traditional treatments. The therapeutic benefits of metal NPs are maintained, while toxicity and environmental impact are reduced using green synthesis methods [12, 13]. Among all the metal NPs, the therapeutic potential and unique properties of copper oxide (CuO) NPs have attracted substantial attention in cancer therapy [14, 15]. Since pre-Vedic times, copper has been essential to human society, supporting conventional medical procedures and daily activities [16, 17].

Co-administering chemotherapeutic drugs with CuO NPs enhances anticancer activity through a synergistic effect. 5-fluorouracil is a common, affordable anticancer agent with various applications, including antibacterial effects. 5-Fu acts as a pyrimidine analog, inhibiting thymidylate synthase, a key enzyme in DNA synthesis. This causes the depletion of thymidine triphosphate (dTTP), leading to DNA damage and stopping the cell cycle in the S-phase. 5-Fu integrates into RNA, disrupting RNA processing and function, leading to cell death by apoptosis [18, 19]. Combining 5-Fu with CuO NPs is a strategic method to enhance therapeutic efficacy, allowing for lower dosages of the anticancer drug, minimizing adverse effects, and improving treatment outcomes.

A localized therapeutic gel delivering CuO NPs and 5-Fu using pectin and xanthan gum may effectively treat cervical cancer. Pectin and xanthan gum have strong mucoadhesive properties, making them suitable for vaginal gel formulations that ensure long-lasting retention and contact with vaginal tissues [20, 21]. Their hydrophilicity helps maintain moisture balance, while their biocompatibility and biodegradability make them non-toxic and suitable for sensitive vaginal environments. The ability to form gels of appropriate viscosity, pectin, and xanthan gum ensures sustained drug release at the target site. This makes the pectin-xanthan gum gel an ideal carrier for localized cervical cancer treatment, enhancing the delivery of CuO NPs and 5-Fu while minimizing side effects [21, 22].

In this work, we synthesized CuO NPs using *T. dioica* seed extract, chosen for its bioactive components like carbohydrates, proteins, and polysterols, which act as natural reducing and stabilizing agents—incorporated them into a therapeutic gel alongside the anticancer drug 5-Fu. This eco-friendly method ensures a sustainable, non-toxic synthesis process. The CuO NPs' properties were evaluated using various methodologies before their incorporation into the gel. The anticancer gel was made with pectin as the gel base and xanthan gum as a co-polymer. No prior studies have reported a gel made from pectin and xanthan gum for anticancer use. The gel was made by adjusting the xanthan gum concentration to assess the sustainable release of the drug. The resulting green-synthesized CuO NPs and 5-Fu-loaded pectin-xanthan gum gel (PXFCu) underwent comprehensive analysis and evaluation. Formulations were tested for organoleptic properties, viscosity, spreadability, and pH. HeLa cell lines were used to test the formulation's anticancer effectiveness. Our research focused on investigating the combined use of this anticancer drug to leverage their potential synergistic effects and provide an improved and less harmful alternative. This integrative approach emphasizes the potential of CuO NPs to improve cancer treatment regimens.

## Materials and methods

### Materials

The required chemicals 5-Fluorouracil (5-Fu) extra pure, 99%, Cupric Nitrate Trihydrate (Cu(NO₃)₂ 3H₂O) extra pure, 98%, glycerol 99%, calcium chloride Dihydrate ACS, 99%, sodium acetate and Dimethyl Sulphoxide (DMSO) ACS, 99.9%, were acquired from SRL Chem. Pvt. Ltd. Mumbai. Pectin (extra pure), xanthan gum (food grade), and sodium hydroxide were obtained from the Fine Chem Lab, Chennai. Hilton agar, Trypsin–EDTA, Dulbecco's Modified Eagle Medium (DMEM), MTT (3-(4,5-dimethylthiazol-2-yl)−2,5-diphenyltetrazolium bromide), were obtained from Hi-Media (Mumbai, India). Bovine fetal serum (FBS) and streptomycin were received from HyClone (USA). Giemsa and Trypan Blue were obtained from (Sigma, USA). All the chemicals were used without any further purification. Milli-Q water was used for the entire experiment.

### Preparation of copper oxide NPs

**Fig. 1:**
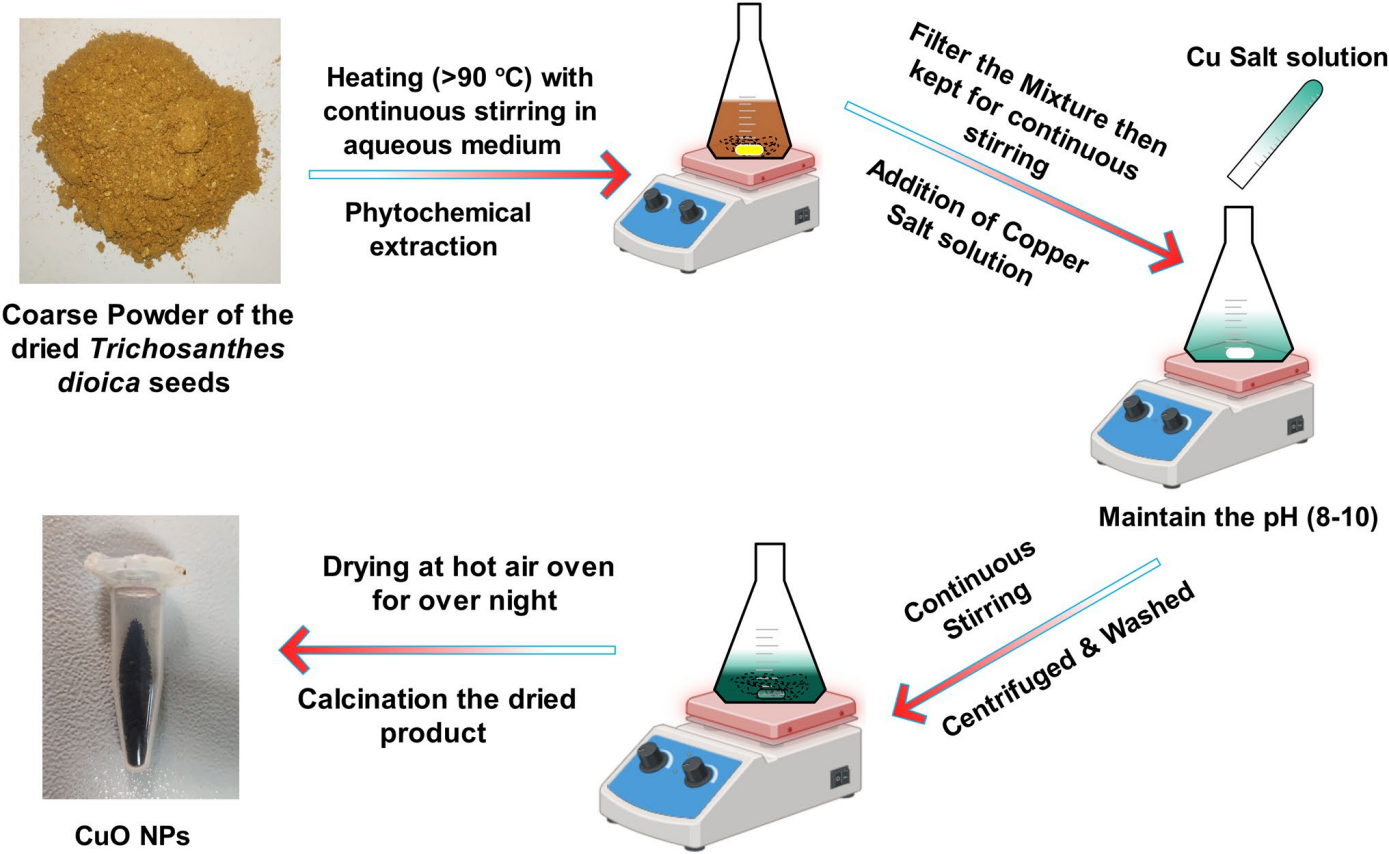
Schematic presentation of preparation of green synthesized CuO NPs

The *T. dioica* dried seeds extract was prepared using the method previously published by our team [23]. Briefly, 20 mL of *T. dioica* dried seeds extract was heated on a magnetic stirrer, maintaining a temperature range of 80–90 °C and a stirring speed of 1000 rpm for 30 min. Subsequently, a 10 mL solution of 0.2 M Cu(NO_3_)_2_ 3H₂O was gradually added dropwise to the extract. The mixture solutions were left to react for 2 h, followed by the slow addition of 0.1 M NaOH solution to achieve an alkaline pH (8–10). This reaction was allowed to proceed for an additional hour. The resulting mixture was left undisturbed to allow NPs sedimentation. The supernatant was removed through centrifugation at 5000 rpm for 10 min. The obtained NPs were cleaned thoroughly using an ethanol–water mixture (1:1) through a process involving sonication and subsequent centrifugation, which was repeated 3–4 times. The particles were then dried in a hot-air oven at 120 °C for 12 h. The CuO NPs dried particles underwent calcination in a muffle furnace at 500 °C for 2 h (Fig. 1).

### Preparation of 0.1% (w/w) CuO NPs and 5-Fu loaded anticancer gel

**Fig. 2:**
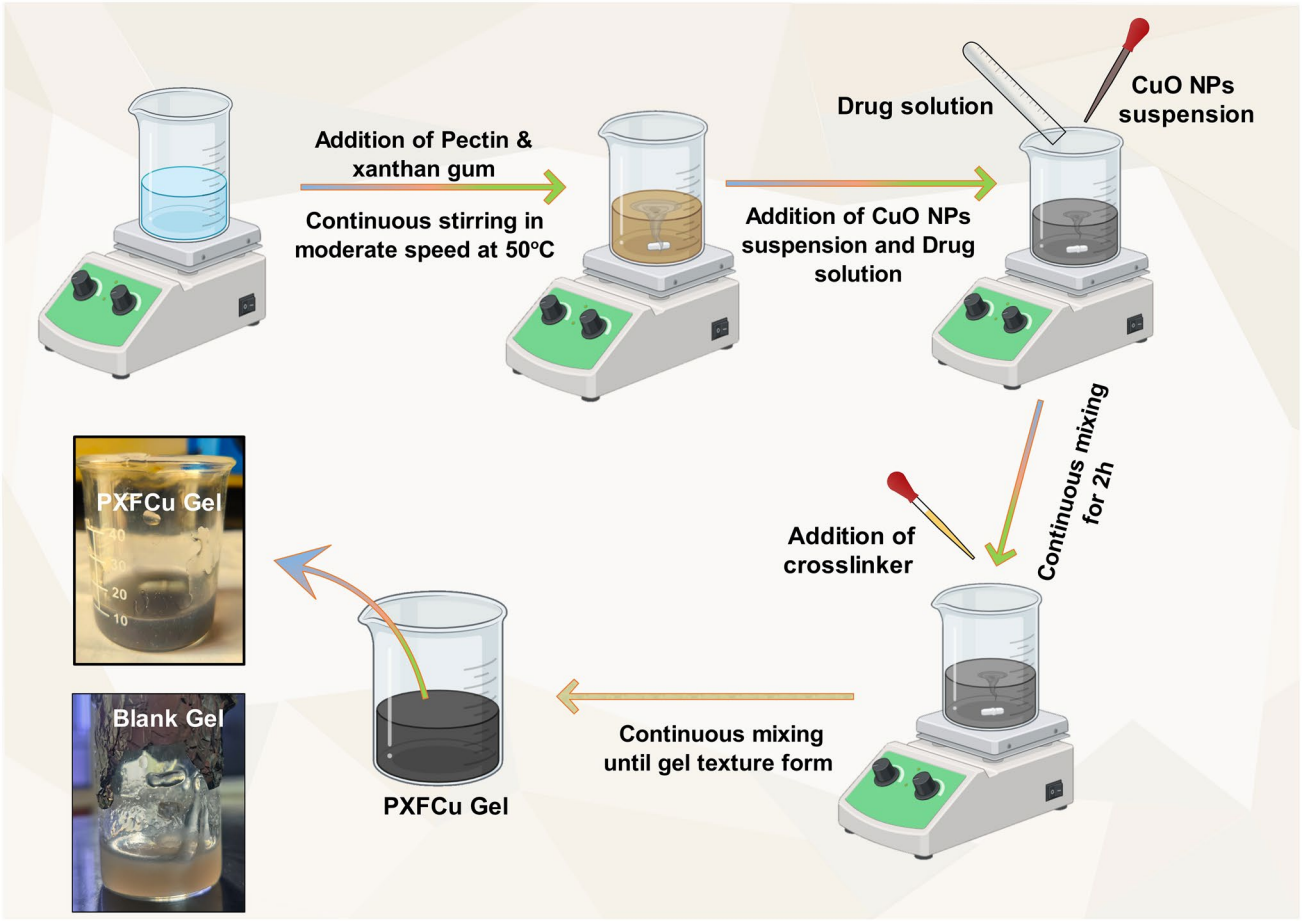
Illustration of the Preparation procedure of 0.1% (w/w) CuO NPs and 5-Fu loaded anticancer gel (PXFCu Gel)

Initially, the pectin (400 mg) and xanthan gum (Table 1) was slowly introduced into warm water (~ 50 °C) to dissolve completely, and the polymer mixture was brought back to room temperature. The accurately dispersed CuO NPs suspension and 5-Fu solution (1 mg/gm) in milli-q water were added to the polymer mixture (0.1% w/w of each of the total weight of the gel). The mixture was allowed to rotate for another 2 h at room temperature at moderate speed (~ 200 rpm). The aqueous cross-linker CaCl_2_ solution (7% w/w) 3 mL was slowly added to the mixture to form a gel. Then, the preservatives (methylparaben, 0.1%) and 0.5 mL of glycerol were added to the gel (Fig. 2). Finally, gels were kept in an amber-colored container and stored in the refrigerator for further characterization. The gels were labelled as PXFCu1, PXFCu2, PXFCu3, PXFCu4, PXFCu5, and PXFCu6.

**Table 1:** Formulation table of PXFCu gel

Formulation code	Pectin: Xanthan gum (w/w)	Drug:CuO NPs (w/w)	Total Milli-Q water (v/v)
PXFCu1	10:1	1:1	20 mL
PXFCu2	10:1.2	1:1	20 mL
PXFCu3	10:1.4	1:1	20 mL
PXFCu4	10:1.6	1:1	20 mL
PXFCu5	10:1.8	1:1	20 mL
PXFCu6	10:2	1:1	20 mL

### Characterization of CuO NPs

The CuO NPs derived from the green synthesis method underwent comprehensive characterization to explore their morphology, surface functionality, size characteristics and etc. Absorption spectra scanning 200 to 800 nm were captured using a UV–vis spectrophotometer (Shimadzu UV-700, Japan). Surface chemistry and functionality analysis of the CuO NPs were performed using Fourier transform infrared spectrophotometry (FTIR, SHIMADZU, Japan). The crystalline state of CuO NPs was evaluated using an X-ray Diffractometer (BRUKER D8 Advance, USA). The Zeta potential was calculated from Malvern/Nano ZS-90 (UK). Morphological evaluation of the produced NPs was studied using High-resolution Transmission Electron Microscopy (JEOL Japan, JEM-2100 Plus). Additionally, elemental mapping was facilitated by an Energy Dispersive X-ray Analyser (EDAX) integrated with the HRTEM.

### Characterization of PXFCu gel

#### Organoleptic characteristics

A comprehensive evaluation was conducted on all 6 PXFCu gel formulations to analyze their physical characteristics, including color, appearance, phase separation, texture, and homogeneity. The aforementioned features were evaluated using visual observations. Examining texture and homogeneity included applying gentle pressure to a tiny quantity of the gels using the thumb and index finger. The assessment of the formulations' texture and homogeneity included an evaluation of their consistency and the absence of coarse particles. Additionally, immediate skin sensation was carefully evaluated, encompassing stiffness, grittiness, and greasiness [24].

#### Drug-excipients compatibility study

The Drug-excipients compatibility study was performed using an FT-IR spectrometer (Bruker) for materials used for the preparation of the gel and optimized gel formulation using FTIR-ATR mode. The spectra were recorded in the range of 4000–400 cm^−1^.

#### Viscosity

The viscosity of all the gels was determined using a Brookfield viscometer (DV-1). The 20 g of gels were taken into the different beakers, and the test was carried out at room temperature. The spindle was rotated at 50 and 100 rpm to evaluate the viscosity of the prepared gels.

#### Spreadability and pH

The spreadability of the PXFCu gels was assessed by determining the expansion of 2 gm of gel in the middle of two horizontal glass plates (20 cm × 20 cm) for one minute. A weight of 25 gm was applied to the upper plate. Each PXFCu gel underwent three separate tests. For the determination of the pH of the gel, 1 gm of each formulation was mixed in 10 mL of deionized water. The pH of these mixtures was then determined using a pH meter.

#### Morphology

HRSEM was used to analyze the morphology of the PXFCu gel. An aluminum sheet was uniformly covered with a thin layer of the PXFCu gel, which was then introduced into the HRSEM chamber after gold sputter coating. The HRSEM offers comprehensive visual representations of the dispersion of CuO NPs throughout the gel matrix.

#### Invitro drug release study

The drug release of 0.1% (w/w) PXFCu gel was examined through a dialysis membrane (Molecular Weight 14,000 Da) technique, maintaining sink condition. Initially, 1 gm of 0.1% (w/w) PXFCu gel (1 mg/gm of 5-Fu) was placed on the dialysis membrane bag and immersed in a beaker with 100 mL of acetate buffer solution (pH 4.2) at 37 ± 1 °C, stirring consistently at 100 rpm. Samples were systematically taken at defined intervals and replaced with an equal volume of fresh buffer to uphold sink conditions. After collection, the samples were assessed using a UV spectrophotometer at 266 nm wavelength to quantify the released drug accurately. The release kinetic models were created regarding the time and % of the drug released.

### Anticancer assay

#### Maintenance and cultivation of cell lines

The NCCS (National Centre for Cell Science, Pune) provided the Human cervical cancer (Hela) cells. 10% FBS, 1 mM L-glutamine solution, streptomycin and penicillin, and 0.2% sodium bicarbonate were added to a DME-supplemented medium with a pH of 7.4. These cells were cultured in this medium. A humidified CO^2^ incubator was used to maintain the culturing conditions at 37 °C. During the experiments, the cells were cultured in a suitable container for the designated duration, either with or without the specified interventions.

#### Cytotoxicity assay (MTT assay)

The cytotoxic effects of green-synthesized CuO NPs, 5-Fu, Blank gel, and PXFCu gel were examined on HeLa cells using the MTT assay. Cells were introduced at 10^4^ cells/well density in 96-well plates for this evaluation. The cells were then permitted to react with variable concentrations of all samples for 48 h at 37 °C in a humidified environment with 5% CO_2_. MTT solution (0.2 g/mL) was introduced after treatment and incubated until violet or purple crystals were observed. After the crystals were dissolved in 200 µL of DMSO, the absorbance was measured at 570 nm using a "(micro-plate reader-Bio-Tek ELX800)." Cell viability was calculated using Eq. (1) [25, 26].

1 $$\% {\text{ Viability }} = \, \left( {OD_{treated \, cells} / \, OD \, c_{ontrol} } \right) \, \times { 1}00$$

Mean *OD *_*treated cells*_ = absorbance of the treated cells, while Mean OD_*control*_ = absorbance of the control [26].

#### Morphological assay (Giemsa stain)

The cells' morphology was seen using Giemsa stain and a bright-field microscope (Dewinter Technologie, India). In short, HeLa cells (10^4^ cells) were set up on 35-mm growth plates specifically intended to enhance cell adherence. Subsequently, the plates were incubated overnight in an incubator at 37 °C with 5% CO_2_ to promote adhesion. Subsequently, the Hela cells were treated with (near IC_50_ concentration) CuO NPs, 5-Fu, blank gel, and PXFCu gel for 48 h at the same condition. After 48 h, the HeLa cells were washed with 3 × PBS and treated with 2% glutaraldehyde for 10 min. Following fixation, the cells underwent rinses through 3 × PBS and were stained with Giemsa solution for 5 min. Subsequently, the dye substance underwent further rinsing using a 3 × PBS solution. The changes in morphology were seen using a Bright-field microscope [27, 28].

### Antibacterial activity

A zone of inhibition assay was carried out using an agar well-diffusion method to assess the antibacterial potency of the green-synthesized CuO NPs, the PXFCu6 gel, and the blank gel. The Mueller–Hinton agar solutions were initially autoclaved at 121 °C and 15 PSI for 15 min. Subsequently, they were poured into sterile glass petri dishes and permitted to settle under aseptic conditions. Wells were created in the agar plates using an 8-mm cork. Afterward, These wells were inoculated with bacterial cultures of *E. coli* (ATCC 8739), a common bacterium associated with urinary tract infections, at a concentration of 10^8^ CFU/mL. Then, 100 µL of blank gel, CuO NPs (10 mg/mL), and PXFCu6 gel were added to each well. The dishes were incubated at 37 ± 0.5 °C for 24 h after moderate agitating for 1 h to facilitate diffusion. After incubation, the plates were analyzed for zones of inhibition around each well, and the diameters of these zones were measured to ascertain the antibacterial efficacy of the experimental substances. The antibacterial properties of the CuO NPs, Blank gel, and the PXFCu6 gel against the *E. coli* strain are compared in this assay.

### Stability assessments

In order to evaluate the stability of the PXFCu6 gel formulation, which contains 5-FU and CuO NPs, over three months, ICH guidelines conducted a stability study. The experiment was conducted at two distinct temperatures: 4 °C ± 0.5 °C (no RH) and 25 °C ± 2 °C (60% RH ± 5% RH). Stability chambers configured at these conditions were utilized to store gel samples. Samples were withdrawn and subjected to a series of tests, including viscosity, pH, appearance, and phase separation, at predetermined intervals (0, 1, and 3 months). The gel's physical, chemical, and therapeutic stability was ensured throughout the storage period by examining the results of these tests under various storage conditions.

### Statistical analysis

The experimental results were analyzed and represented by the mean value of the standard deviation using Graph Pad PRISM, USA, the experiments are conducted thrice.

## Results and discussion

### Green synthesis of CuO NPs

The CuO NPs were synthesized using a green method. Initially, the *T. dioica* dried seeds powder was boiled to create the extract, releasing its different phyto-constituents into the aqueous medium. These natural compounds played a crucial role in reducing copper nitrate to CuO NPs and stabilizing the NPs during formation (Fig. 1) [12, 29]. After the synthesis, dark green sediment was formed, turning into dark brown particles after drying and calcination. The implementation of green synthesis methods offers a wide range of benefits, the most prominent of which is a substantial decrease in energy consumption and production costs associated with NPs manufacturing. Furthermore, using phytochemicals accomplishes the dual objectives of catalyzing and stabilizing these NPs, exhibiting a significantly reduced toxicity compared to synthetic alternatives [30]. This intrinsic quality promotes a more secure methodology and facilitates adjustments and modifications in the composition of metal NPs. Moreover, the intriguing existence of few phytochemicals on the metal NPs' surface offers excellent potential for enhanced interactions with a wide range of organic compounds, including drugs, biomolecules, lipids and polymers [31]. This distinctive characteristic acts as a catalyst to improve their efficacy and adaptability, making them exceptionally well-suited for a broad spectrum of biological applications. The NPs' intrinsic adaptability allows for customization and refinement, broadening their potential applications across diverse domains via meticulous alterations [32–34].

### Characterisation of CuO NPs

#### Optical properties of CuO NPs

**Fig. 3:**
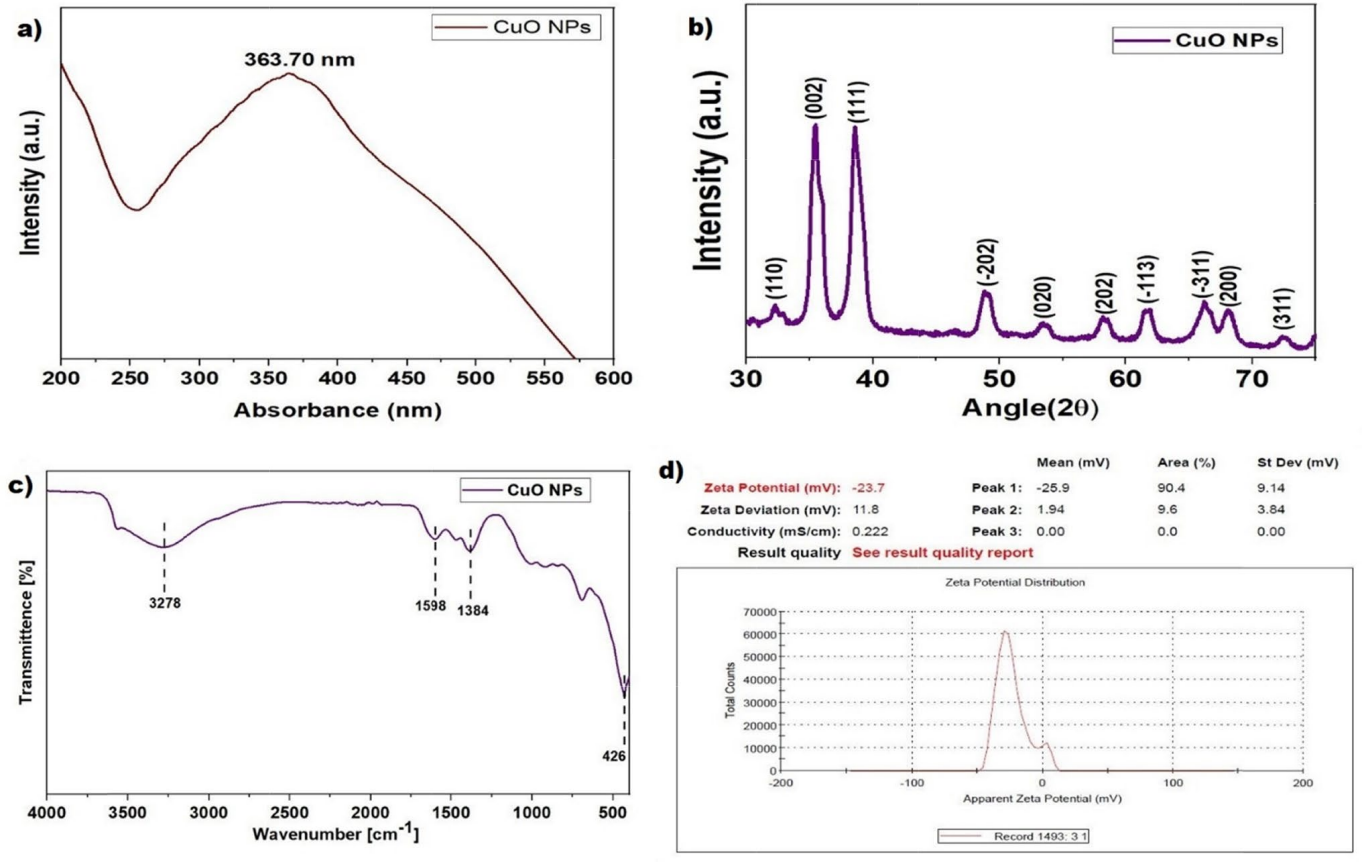
(**a**) The optical spectrum of CuO NPs; (**b**) The XRD spectrum of CuO NPs; (**c**) The FTIR spectrum of CuO NPs; (**d**) the zeta potential of CuO NPs

A comprehensive optical property analysis was conducted utilizing a UV–Vis spectrometer to examine green synthesized CuO NPs. The study measured absorbance across a wavelength spectrum from 200 to 800 nm, with the findings graphically represented. The investigation revealed the maximum wavelength (λ_max_) of CuO NPs is 363.7 nm (Fig. 3a). Similarly, Nzilu et al. found the maximum wavelength of *Parthenium hysterophorus* whole plant aqueous extract-mediated CuO NPs is 340 nm [35]. Also, Jadhav et al. found the λ_max_ of *Malus domestica* leaf extract-mediated CuO NPs is 335 nm [36]. In this study, the wavelength of CuO NPs increases toward a slightly higher wavelength, which can decrease the energy bandgap, which in turn leads to an increase in absorption and conductivity. This enhanced absorbance can produce higher optical conductivity, particularly in ultraviolet [37, 38].

#### FTIR analysis of CuO NPs

The surface chemistry of CuO NPs was investigated using ATR-FTIR, revealing the presence of distinctive functional groups. In Fig. 3c, the FTIR spectrum showcased prominent peaks at 3278 cm^−1^, 1598 cm^−1^, 1384 cm^−1^, and 426 cm^−1^. The peak at 3278 cm^−1^ corresponds to the stretching vibration of the O–H group of carboxylic acid, indicative of surface functionalization (Table 2). Additionally, peaks at 1598 cm^−1^ and 1384 cm^−1^ signify the bending vibrations of the N–H group of primary amine and the C–O–H group, respectively, suggesting the involvement of these functional groups in the NP's surface chemistry[35, 39, 40]. Notably, a significant peak at 426 cm^−1^ was observed, attributed to the presence of Cu–O bands, affirming the formation of CuO NPs[41, 42]. The discernible presence of these organic functional groups underscores the green synthesis approach employed, utilizing plant extracts rich in phytochemicals. These functional groups, derived from the plant extract, contribute to the surface modification of CuO NPs and serve as catalysts during the green synthesis process. This comprehensive understanding elucidates the intricate interplay between phytochemicals and NPs surface chemistry, emphasizing the sustainable and eco-friendly nature of the green synthesis method [12, 23].

**Table 2:** The IR interpretation of the CuO NPs

Wavenumber (cm⁻^1^)	Functional group	Peak assignment/observation	Significance
3278	O–H (carboxylic acid)	Stretching vibration of O–H group	Indicative of surface functionalization through carboxylic acid groups
1598	N–H (primary amine)	Bending vibrations of N–H group	Suggests the involvement of primary amine in surface chemistry
1384	C–O–H (hydroxyl group)	Bending vibrations of the C–O–H group	Indicates the presence of hydroxyl functional groups on the surface
426	Cu–O	Stretching vibrations of Cu–O bond	Confirms the formation of CuO

#### XRD analysis CuO NPs

The structural features of CuO NPs, synthesized through environmentally sustainable techniques, were meticulously examined via X-ray diffraction analysis, as depicted in Fig. 3b. The XRD pattern revealed distinct peaks at 2θ values with correspondence lattice planes of 32.23° (−110), 35.42° (002), 38.55° (111), 48.76° (−202), 53.43° (020), 58.26° (202), 61.69° (−113), 66.17° (−311), 68.09° (200), and 72.49° (311) respectively. These identified peaks closely match the characteristic pattern of a monoclinic crystal structure (JCPDS: 45–0937). This congruence indicates the successful synthesis of CuO NPs exhibiting a monoclinic phase. To further elucidate the structural properties, the Debye-Scherer equation (Eq. 2) was employed to calculate the crystalline size of the synthesized CuO NPs. This calculation yielded an estimated crystal size of approximately 8.85 nm [43–45].

2 $$D=\frac{k\uplambda }{\beta cos\theta }$$

Here, D is crystalline size, where k is 0.98, λ is the wavelength, and β denotes full width at half maximum.

#### Zeta potential of CuO NPs

The Malvern Zetasizer was used to determine the zeta potential of the CuO NPs. The measured average Zeta potential was −23.7 mV (Fig. 3d), indicating the presence of negative surface charges on the CuO NPs in the aqueous dispersion [42]. A negative zeta potential means that the CuO NPs are within the range that ensures colloidal stability. This suggests a reduced probability of the CuO NPs clumping together and settling to the bottom of the solution [46]. These properties facilitate enhanced dispersion, cellular absorption, and targeted drug delivery, improving safety and effectiveness.

#### Morphological analysis of CuO NPs

**Fig. 4:**
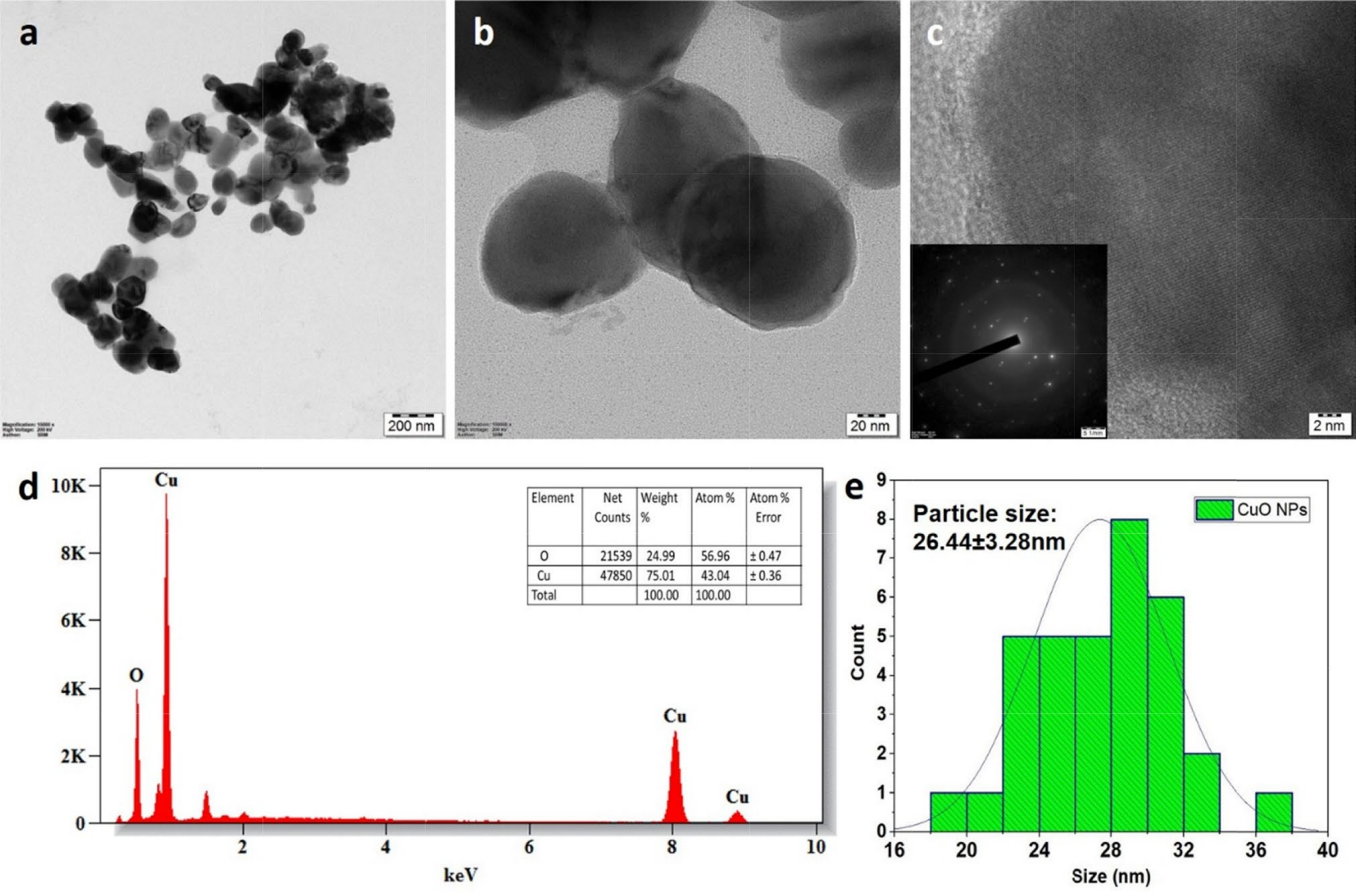
Morphology of the CuO NPs through TEM (**a**) CuO NPs in lower resolution (scale 200 nm); (**b**) CuO NPs in higher resolution focussing on single NPs (Scale 20 nm); (**c**) CuO NPs in higher resolution focussing on single NPs surface along with SEAD pattern; (**d**) the elemental analysis of CuO NPs through EDX. (**e**) The distribution of particle size of CuO NPs

A thorough examination of the morphology of CuO NPs was conducted through TEM analysis. The CuO NPs were accurately deposited onto a carbon-coated copper grid and subjected to TEM observation. Figures 4a–c vividly illustrate the images captured during this analysis. The TEM analysis unveiled that the CuO NPs synthesized through the green technique predominantly exhibit a spherical morphology characterized by particle sizes ranging from 24 to 32 nm. Using Image J software, the average particle size was estimated to be 26.44 ± 3.28 nm (Fig. 4e). Notably, these measured particle sizes were larger than the crystalline sizes determined through XRD, which were approximately ~ 8.8 nm. This difference in size measurements can be attributed to the complex interplay of factors during the synthesis process. Specifically, it's attributed to the aggregation of crystallites throughout the formation of the colloidal particle dispersion containing calcinated or dried NPs. Such amalgamation phenomena often lead to variations in particle size observed in different analytical techniques. Moreover, the elemental composition of the CuO NPs was confirmed through energy-dispersive X-ray spectroscopy (EDS), as depicted in Fig. 4d. The EDS spectra explicitly demonstrate the presence of copper and oxygen, further validating the composition and elemental purity of the synthesized material.

### Preparation of PXFCu anticancer gel

Pectin, a natural polysaccharide, possesses exceptional biocompatibility, gel-forming capabilities, and mucoadhesive properties, guaranteeing therapeutic agents' sustained release and protracted retention at the applied site [47, 48]. On the other hand, xanthan gum, a polysaccharide, improves the gel's stability and viscosity, thereby establishing a homogenous and durable matrix for incorporating 5-Fu and CuO NPs [49, 50]. During the process of gel preparation, pectin undergoes chemical crosslinking, which is helped by ionic crosslinkers. This crosslinking improves the stability and functioning of the final gel [51, 52]. The gel formation of pectin is closely connected to its molecular structure and interactions, which are affected by environmental conditions such as pH, temperature, and ion concentration. Pectin gels are usually created by a process called ionotropic gelation. In this process, divalent ions like calcium function as crosslinkers, connecting nearby pectin chains by attaching to the carboxyl groups of galacturonic acid residues. The gelation process is carefully controlled by pH and ionic strength parameters. Acidic conditions promote the protonation of pectin molecules and facilitate the binding of calcium ions [53, 54]. This thorough comprehension emphasizes pectin's applicability as an ingredient that can form a gel and can be used for numerous industrial and biological applications. On the other hand, Xanthan gum is well-known for its efficient stabilizing and thickening properties. Xanthan gum consists of extended carbohydrate chains that create a network capable of efficiently trapping water molecules, hence greatly increasing the viscosity of solutions and formulations [55]. These two polymers were chosen for the gel formulation to deliver the CuO NPs and drug 5-Fu. The concentration of the drug and CuO NPs was set at 0.1% w/w of the total weight of the gel.

The xanthan gum was utilized to enhance the characteristics of pectin in the formulation of the anticancer gel. After the literature surveys, 2% w/w pectin and xanthan gum were taken at different concentrations, ranging from 10 to 20% of the weight of pectin, to evaluate their effectiveness in enhancing the gelation process in conjunction with pectin. The gel was created by dissolving pectin and xanthan gum in warm water in precise amounts. Subsequently, the aqueous drug solution and CuO NPs suspension were introduced into the polymer solution, followed by the addition of a CaCl_2_ solution (Fig. 4). The calcium ions (Ca^2+^) of CaCl_2_ interact with the carboxyl groups in the polymers, triggering the crosslinking of neighboring pectin chains via a process called "egg-box" production [56]. This contact facilitates the formation of connections between pectin molecules, creating a three-dimensional network. The resulting interconnected pectin structure, reinforced by the thickening properties of xanthan gum, traps water molecules, 5-Fu, and CuO NPs inside the polymer matrix [57, 58]. The 5-Fu and CuO NPs were added 0.1% of the total weight of the gel. This combination could promote adherence to the vaginal mucosa and sustain the release of the drug and metal NPs at the cervical region.

### Organoleptic characteristics of PXFCu gel

Each of PXFCu gel formulation underwent rigorous testing to evaluate its physical attributes, encompassing appearance, color, texture, phase separation, and homogeneity. The initial observation revealed a distinct translucent black appearance, a characteristic attributed to the presence of CuO NPs within the formulation (Table 3). This black hue, imparted by the CuO NPs, is a visual indicator of their incorporation and concentration within the gel matrix. Upon further examination, the formulations displayed a smooth and uniform texture devoid of any discernible coarse particles. This uniformity in texture is essential for ensuring consistent application and delivery of the gel. A critical aspect of formulation stability is the absence of phase separation, which was meticulously evaluated over an extended period. Despite being retained for 90 days in cold and room temperature in an amber color container, no evidence of phase separation was observed, indicating the robustness and homogeneity of the gel formulation. This absence of phase separation underscores the formulation's efficacy in maintaining its integrity and uniform distribution of active ingredients over time, ensuring consistent performance and efficacy upon application.

**Table 3:** Physicochemical characteristics of the PXFCu gel

Formulation code	Appearance	Homogeneity	pH	Spreadability (cm)
PXFCu1	Translucent black	Good	4.1 ± 0.13	6.9 ± 0.16
PXFCu2	Translucent black	Good	4.2 ± 0.07	6.5 ± 0.12
PXFCu3	Translucent black	Good	4.5 ± 0.11	6.5 ± 0.16
PXFCu4	Translucent black	Very Good	4.2 ± 0.11	6.4 ± 0.22
PXFCu5	Translucent black	Good	4.5 ± 0.14	6.4 ± 0.08
PXFCu6	Translucent black	Very Good	4.6 ± 0.18	6.1 ± 0.16

(n = 3), Data represented as mean ± S.D

### Drug-excipients compatibility study

**Fig. 5:**
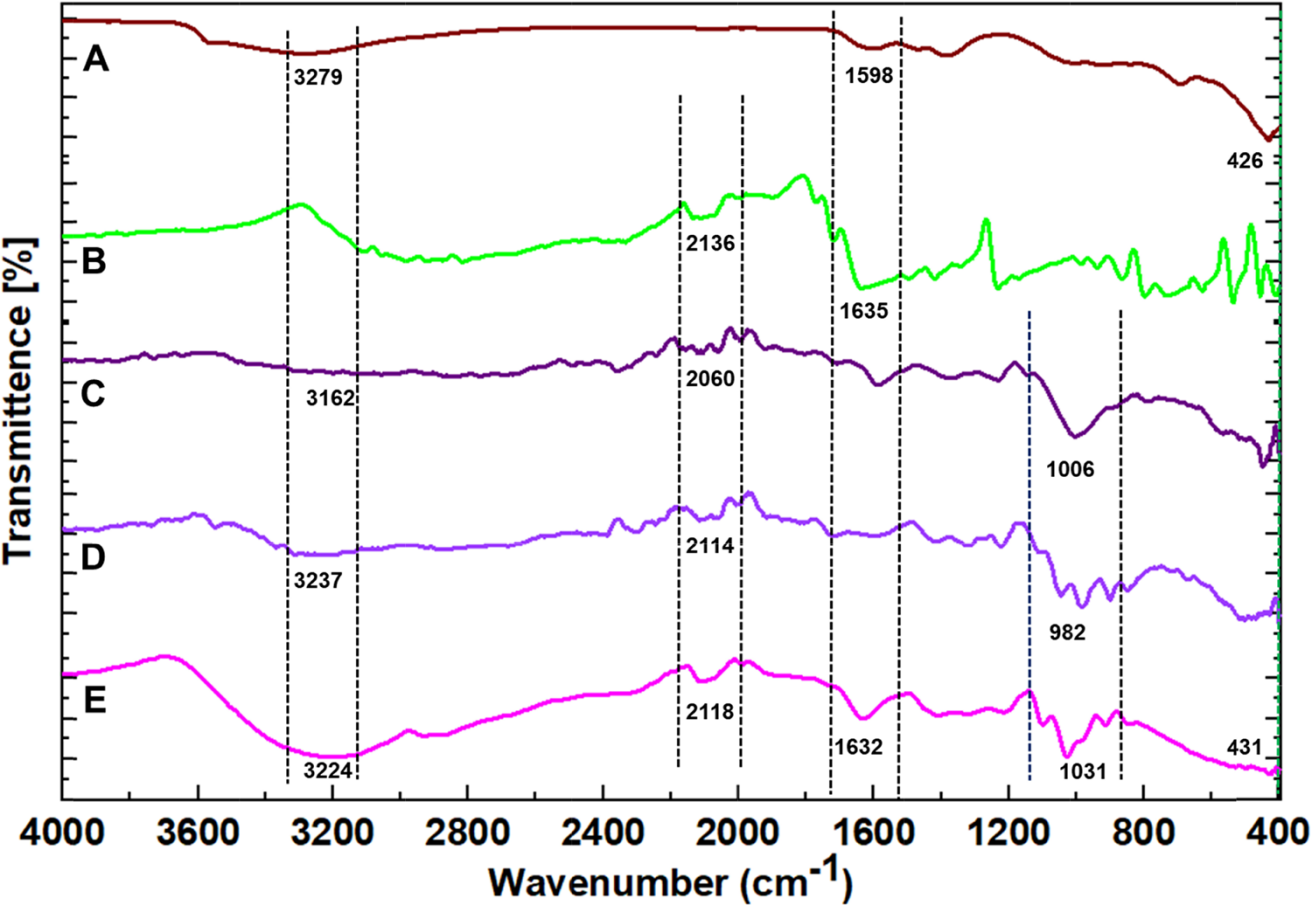
(**a**) IR spectrum of CuO NPs; (**b**) IR spectrum of 5-Fu; (**c**) IR spectrum of xanthan gum; (**d**) IR spectrum of pectin; (**e**) IR spectrum of PXFCu gel

The drug excipient compatibility investigation used FTIR analysis, a prevalent method for evaluating chemical interactions among various constituents. Figure 5A–E exhibited several discernible characteristics in the spectra. The presence of carboxylic acid groups, possibly arising from components such as xanthan gum, pectin, and green-synthesized CuO NPs, is suggested by the curvature peak between 3300 and 3100 cm^−1^ in spectra A, C, D, and E, which corresponds to O–H stretching. Furthermore, the significant peaks seen at wavelengths between 2200 and 2000 cm^−1^ in spectra B, C, D, and E were identified as resulting from the stretching of C≡C bonds. These peaks are likely connected with specific components, such as aromatic structures. In addition, the appearance of particular peaks at 1635 cm^−1^ in spectrum B and 1632 cm^−1^ in spectrum E indicated the occurrence of N–H bending of primary amines. This observation may be defined as the drug molecule itself in the gel formulation. Furthermore, the existence of C–O stretching, a characteristic of primary alcohols often seen in polysaccharides such as xanthan gum and pectin, was revealed by the identical overlapping peaks observed between 1100 to 900 cm^−1^ in spectra C, D, and E. Two comparable peaks were seen at about 450 cm^−1^ in spectra A and E, indicating the existence of metal NPs. These peaks are attributed to Cu–O bands. The presence of CuO NPs in the gel formulation was verified. Crucially, the lack of notable disparities in the spectra of the drug (5-Fu) and other gel components in spectrum E indicates no significant interactions throughout the process of producing the gel [59–62]. The finding is substantiated by the persistent existence of unique peaks in all spectra, suggesting the drug's compatibility with the other components in the PXFCu anticancer gel.

### Determination of spreadability and pH

The spreadability of PXFCu gels was measured in centimeters and found to vary from 6 to 7 cm presented in Table 3. The gel's composition significantly influenced its spreadability. The interplay of the constituents, such as pectin and xanthan gum, was important in determining the gel's ability to disperse efficiently[63]. Simultaneously, the pH values of the gels were assessed to determine their suitability for vaginal application. The resultant pH of the gel indicates it may be compatible with the vaginal pH. This also highlights the significance of comprehending how the interaction of ingredients, like pectin and xanthan gum, affects both the ability of the gel to spread and its suitability for its application [64, 65].

### Morphology of the PXFCu gel

**Fig. 6:**
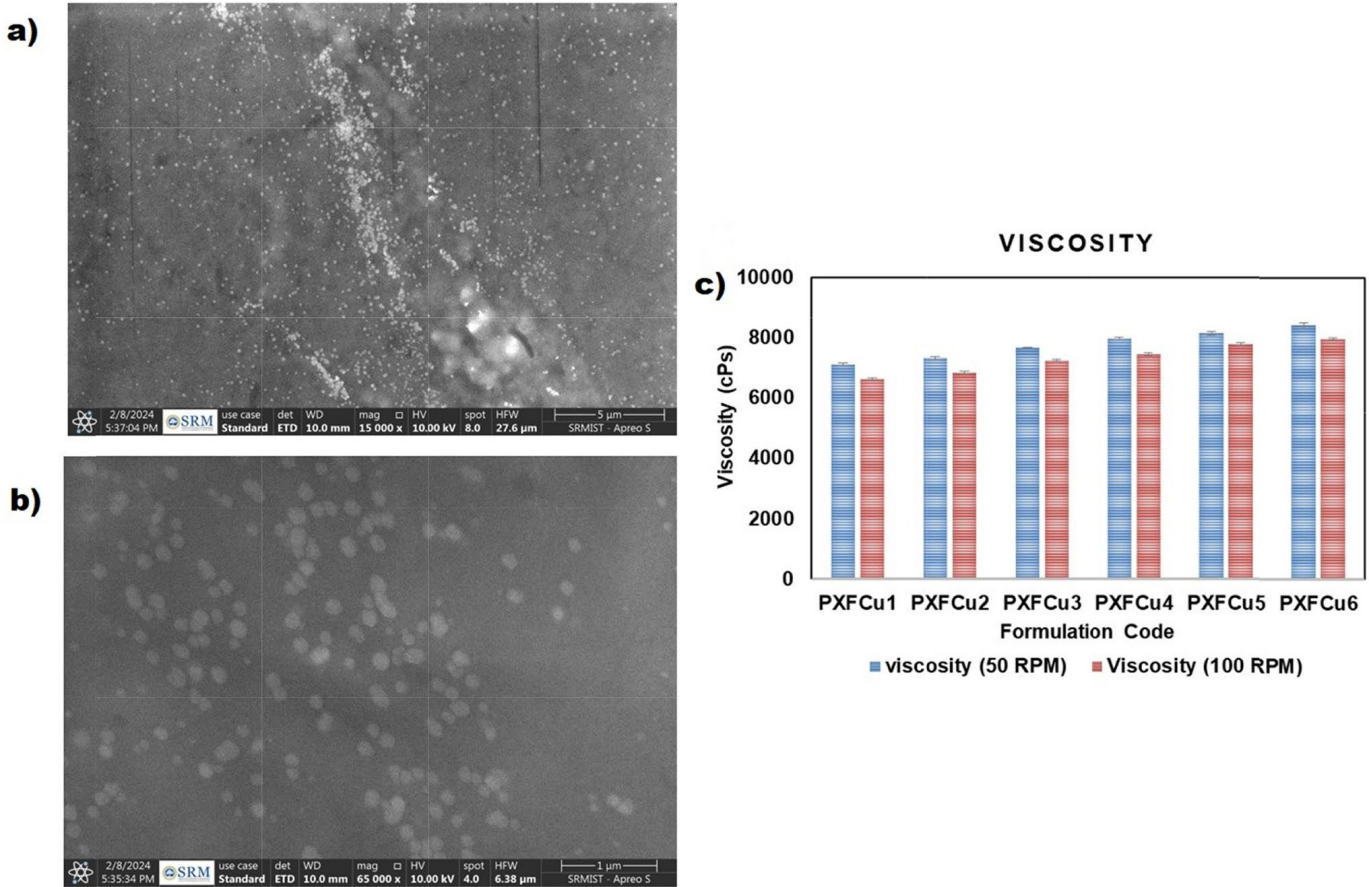
Morphology of PXFCu gels under HRSEM (**a**) The PXFCu gel under 15,000 × magnification (scale 5 µm); (**b**) The PXFCu gel under 65,000 × Magnification (scale 1 µm); (**c**) the graphical representation of the all PXFCu gels viscosity, Data represent as mean ± S.D (n = 3)

The HRSEM (Thermosceintific Apreo S) was utilized to evaluate the morphology of the gel. The gels were deposited onto aluminum foil and desiccated under vacuum conditions to produce a PXFCu gel thin film. The findings, as shown in Figs. 6a and b in different magnifications, demonstrate the successful distribution of CuO NPs without any noticeable big coagulation of CuO NPs on the gel. The effective dispersion of all gel constituents is apparent, as shown by the lack of polymer agglomerations.

### Determination of viscosity

The viscosity measurements for the formulations were performed at rotational speeds of 50 and 100 rpm, with a one-minute time interval for each gel, as shown in Fig. 6c. The viscosity of gels containing bioactive compounds was measured between 7106 ± 22 and 8426 ± 40 cP at 50 rpm and between 6631 ± 34 and 7984 ± 93 cP at 100 rpm. The viscosity of the gel formulations showed a noticeable increase, which may be ascribed to changes in the amount of xanthan gum in the PXFCu gels. This implies that when the concentration of xanthan gum rises, the viscosity of the gel similarly increases. The viscosity of PXFCu gels is influenced by the concentration of xanthan gum due to its distinct rheological characteristics. The concentration of xanthan gum significantly influences the density and strength of the gel network. Raising the concentration of xanthan gum intensifies the entanglement and interaction among polymer chains, leading to a more robust gel structure. The increased level of contact results in an elevated viscosity, as seen by the viscosity tests.

### Invitro drug release from PXFCu gel

**Fig. 7:**
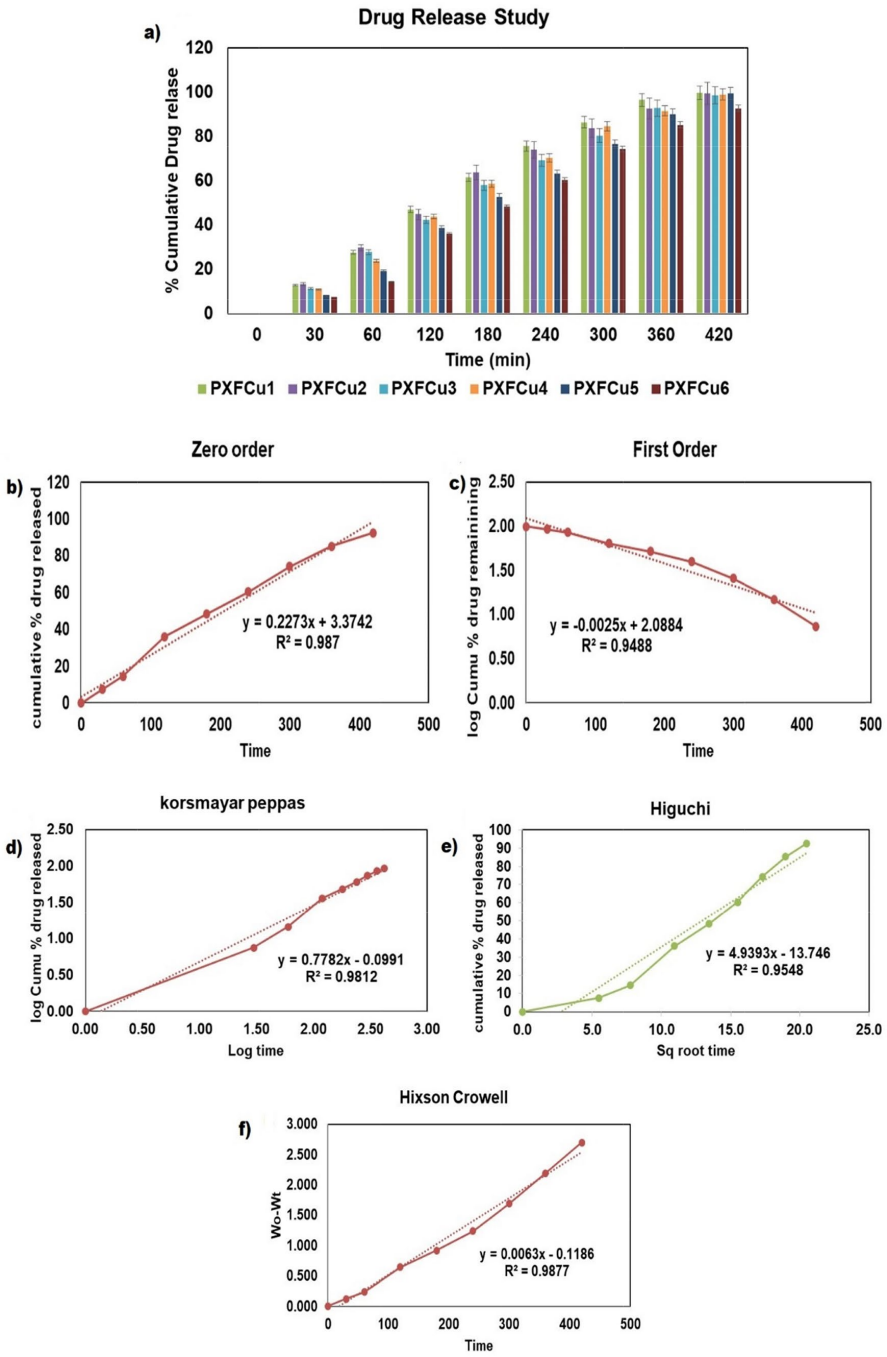
The drug release profile of six PXFCu anticancer gel formulation (**a**); The zero-order kinetics of PXFCu6 gel (**b**); The first order kinetic of PXFCu6 gel (**c**); korsmayar peppes kinetic model of PXFCu6 gel (**d**); Higuchi kinetic model of PXFCu6 gel (**e**); Hixcon crowell kinetic model of PXFCu6 gel (**f**)

The drug-release investigation utilized the dialysis membrane method, a frequently used *in-vitro* approach to simulate drug release over a semi-permeable barrier and preserve the sink condition. During the initial phase of the release experiments, it was noticed that the gel formulations began to release the 5-Fu after they were placed in the release media (Fig. 7A). Distinctive release profiles were observed for each tested formulation, specifically PXFCu1, PXFCu2, PXFCu3, PXFCu4, PXFCu5, and PXFCu6. The first three formulations, PXFCu1, PXFCu2, and PXFCu3, demonstrated a rapid drug release, with more than 60% of the 5-Fu being released within the first 180 min. Notably, PXFCu4 and PXFCu5 exhibited a consistent and progressive drug release, with about 99% of the 5-Fu being released between 360 to 420 min. Significantly, PXFCu6 demonstrated a consistent pattern of drug release from the beginning, persistently releasing the drug for 420 min. The observed release data highlights the crucial influence of the xanthan gum content in controlling the release of the medication from the gel matrix for all the gel formulations. Elevating the concentration of xanthan gum resulted in developing a more complex and complicated three-dimensional network structure inside the gel matrix. Consequently, the enhanced network structure hindered the spread of the drug molecules, leading to a sustained release rate.

Several kinetic models were examined to clarify the release kinetics of PXFCu6, and the regression coefficient (R^2^) was calculated to determine the most accurate fit (Fig. 7B–F). The drug release profile of PXFCu6 showed a perfect fit with the Hixson-Crowell kinetic model, resulting in a high regression value of 0.9877. This indicates that the erosion or dissolution of the gel matrix primarily controls the drug release from PXFCu6. The nature of both polymers used in PXFCu6 helps maintain the integrity of the gel structure. This sustained contact allows for a slow and controlled release of the drug. As a result, the drug release rate decreases over time because the available surface area for release decrease. Due to its prolonged release profile and ability to effectively control the release of the drug from the gel matrix, PXFCu6 emerged as the optimal candidate for further evaluation in anticancer assays.

#### Anticancer assays

**Fig. 8:**
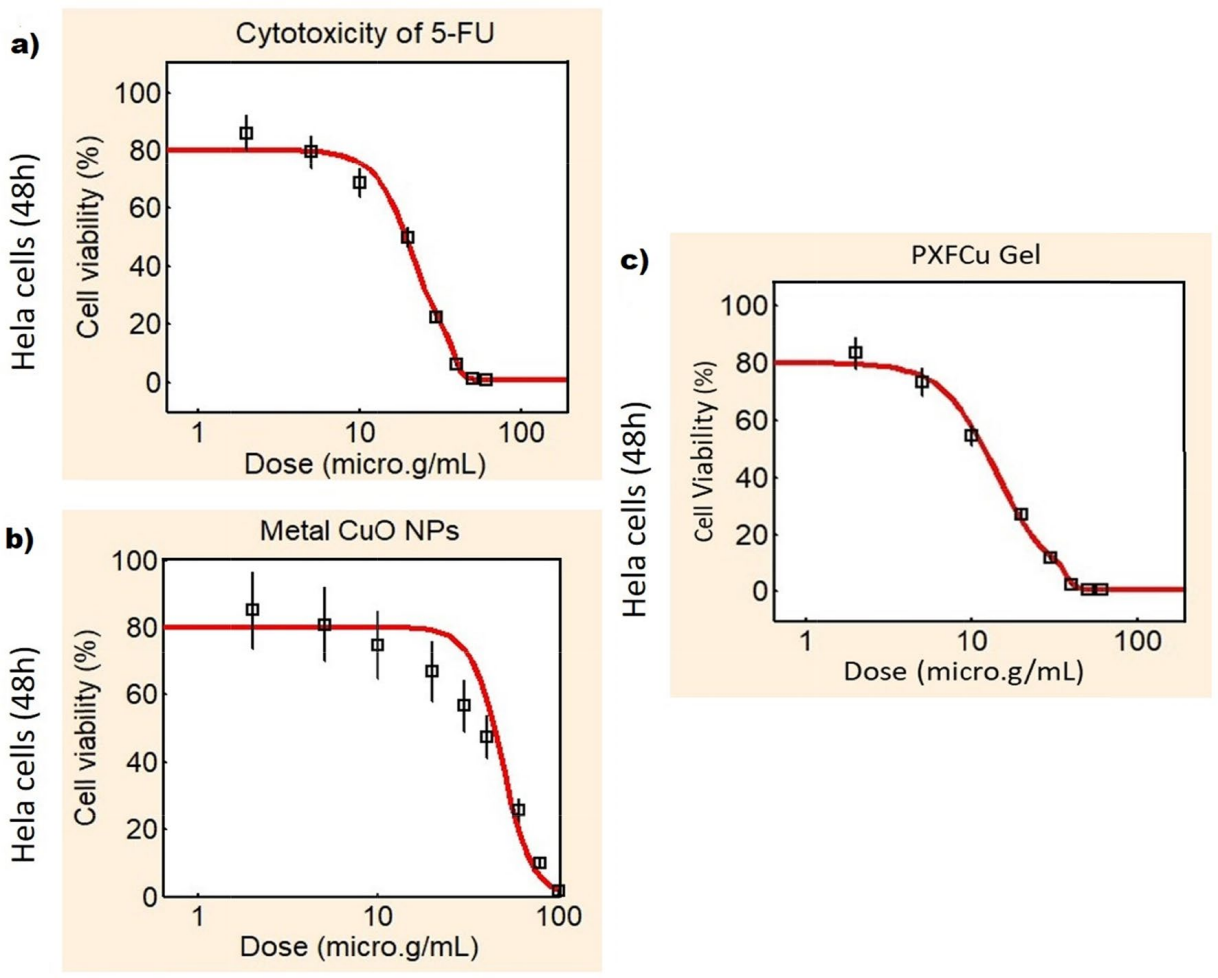
**a** Dose–response curve of 5-Fu against HeLa cell line; **b** Dose–response curve of CuO NPs against HeLa cell line; **c** Dose–response curve of PXFCu6 Gel against HeLa cell line; the dose–response curve was constructed by Dr. Fit software. [Data represent as mean ± S.D (n = 3)]

The MTT test was used to assess the cytotoxicity of *T. dioica* dried seeds extract mediated CuO NPs on HeLa cells after 48 h of treatment, as seen in Fig. 8B. The cells were treated for 48 h with various concentrations of CuO NPs ranging from 2 to 100 μg/mL. The findings demonstrated a reduction in cell viability directly proportional to the dosage. The IC_50_ concentration of CuO NPs on HeLa cells was 42.8 ± 0.24 μg/mL, indicating, indicatingficant anticancer activity on human cervical cancer cells. Likewise, other research groups have synthesized CuO NPs using environmentally friendly methods and assessed their effectiveness against various cancer cell lines to determine their potential as anticancer agents [32]. Mohammad et al., produced CuO NPs using *Vitex negundo* and evaluated their anticancer properties by testing them on two types of cancer cells, HepG2 and Hela. The IC_50_ value for HepG2 cells was 49 µg/mL, whereas for Hela cells, it was found to be 82 µg/mL [66]. Elemike et al. developed CuO NPs, with 63.64% potential for inducing apoptosis in HeLa cells at a concentration of 100 µg/mL [67]. In another study, Dulta et al. found that 77 nm-sized green CuO NPs produced using *Carica papaya* leaf extract showed anticancer properties on HeLa cells, with an IC_50_ value of 139.6 μg/mL measured [68]. Similarly, Rajamma et al. developed CuO NPs using a plant extract from *Nilgirianthus ciliatus* and exhibited LC_50_ values of 85.58 and 81.57 µg/mL for the MCF-7 and A549 cell lines, respectively [69]. The primary mechanism behind the anti-cancer properties of CuO NPs is their capacity to induce the production of reactive oxygen species (ROS) inside HeLa cells, resulting in oxidative stress. Increased quantities of ROS may cause harm to several biological components, such as DNA, proteins, and lipids [70, 71]. In addition, studies have shown that CuO NPs may enhance the levels and functionality of essential proteins involved in programmed cell death, such as caspase-3 and p53. Caspase-3 is an essential mediator of apoptosis, playing a critical role in the fragmentation of different cellular components, ultimately resulting in the disintegration of the cell. The tumor suppressor protein p53 is crucial in controlling the cell cycle and inducing programmed cell death (apoptosis) in response to cellular stress and DNA damage [72]. In addition, CuO NPs cause a considerable disruption of the mitochondrial membrane potential in cancer cells. This hampers the organelle's capacity to generate ATP, the cellular energy unit. The synergistic impact of ROS production, activation of apoptotic proteins, and impairment of mitochondrial function culminate in the efficient induction of apoptosis in cancer cells [71, 73].

In comparison, the 5-Fu was treated with Hela cells with different concentrations, and the IC_50_ concentration was 19.3 ± 0.492 μg/mL at 48 h (Fig. 8A). Research by Gen et al., found the IC_50_ concentration of 5-Fu of 43.34 ± 2.77 µM on Hela cells [74]. Mavrikou et al. reported that 5-Fu can increase the Caspase-3 enzymes up to 48 h of incubation, leading to apoptosis [75]. The cytotoxicity of the PXFCu6 gel was assessed using various concentrations and the IC_50_ value found 11.82 ± 0.219 μg/mL (Fig. 8C). On the other hand, the blank gel did not demonstrate any harmful impact on HeLa cells. The results suggest that incorporating the metal and drug in a 1:1 ratio in the PXFCu6 gel dramatically increases its effectiveness. The gel combination exhibited a potency of about 3.62 times greater than that of *T. dioica* dried seeds extract mediated CuO NPs alone and approximately 1.63 times higher than free 5-Fu alone. The interaction between CuO NPs and 5-Fu in the PXFCu6 gel results in a more potent cytotoxic impact on HeLa cells. Significant anticancer effects were observed in the PXFCu gel at comparatively low concentrations of CuO & 5-Fu against HeLa cells, essential for establishing the optimum dosage for local delivery. Lower drug concentrations enhance patient compliance and mitigate the risk of adverse side effects.

#### Morphological assessment of HeLa cells

**Fig. 9:**
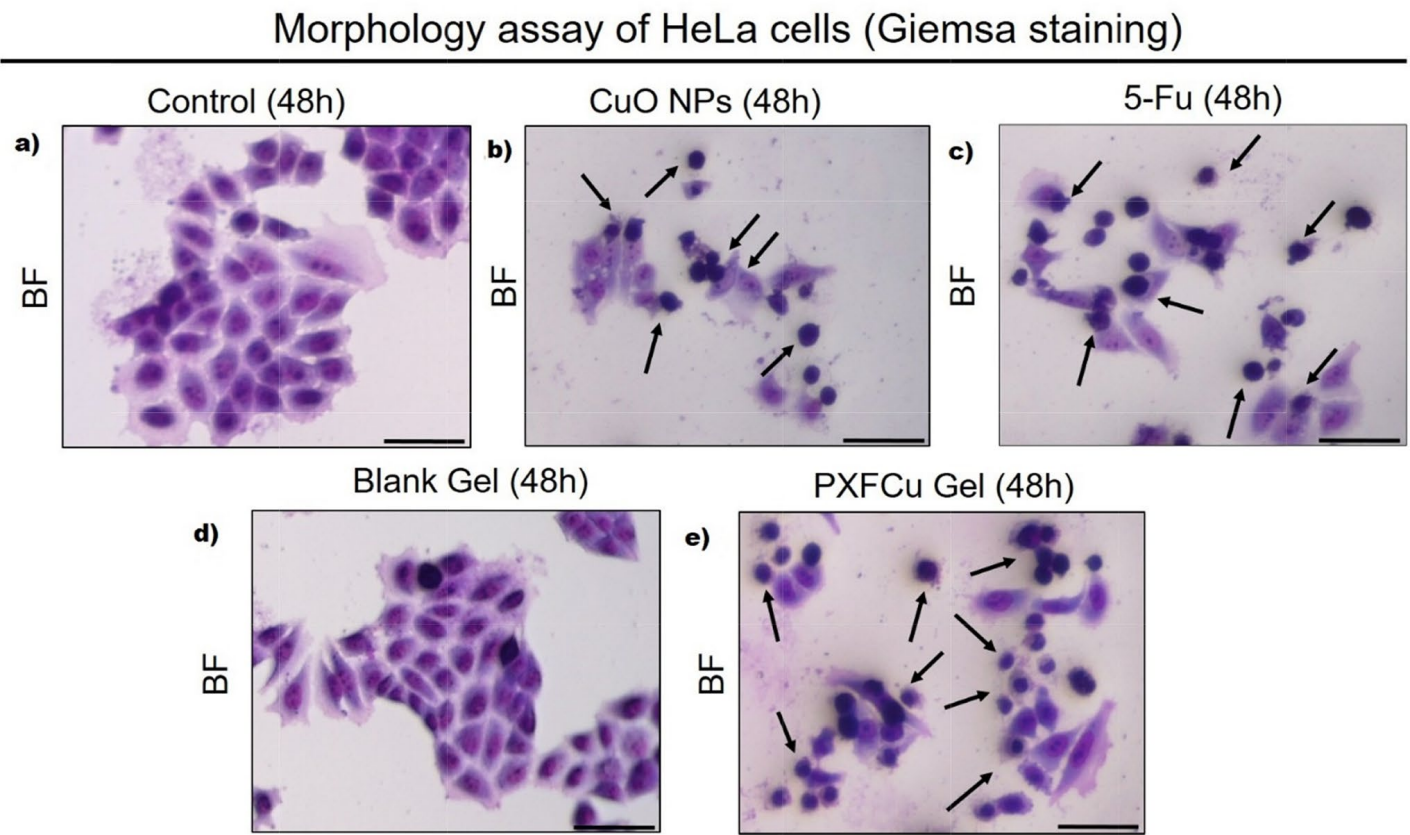
The morphological alterations of HeLa cells following treatment were visualized in a bright-field microscope by staining them with Giemsa (Scale bar: 50 μm). **a** Control; **b** Treated with green synthesized CuO NPs; **c** Treated with 5-Fu; **d** treated with blank Gel; **e** treated with the PXFCu6 anticancer Gel

The morphology of HeLa cells was examined using Giemsa staining under a bright-field microscope. The cells were treated with various samples at concentrations near the IC_50_ and then stained with Giemsa solution (Fig. 9). The control cells displayed the typical morphology of HeLa cells, with distinct shapes and correctly oriented nuclear components (Fig. 9A). After treatment with green synthesized CuO NPs (40 μg/mL), 5-Fluorouracil (20 μg/mL), and PXFCu6 gel (10 μg/mL), notable changes in cell morphology were observed. In particular, apoptotic HeLa cells were visible in Fig. 9B, C and E. These apoptotic cells exhibited characteristic features such as shrinkage and a spherical shape. Early apoptotic cells showed cytoplasmic shrinkage, nuclear condensation, and rounding. Arrowheads indicated apoptotic cells. Figure 9D depicted HeLa cells treated with the blank gel, showing no apoptosis signs. This suggests the blank gel does not induce cytotoxic effects in HeLa cells. The absence of cytotoxicity in the blank gel-treated cells supports the conclusion that the observed effects in the other samples were due to the active components (CuO NPs and 5-Fu) and not the gel matrix. The Giemsa staining results provided clear visual evidence of the morphological changes associated with apoptosis in treated HeLa cells. This observation underscores the enhanced cytotoxic effect of the PXFCu6 anticancer gel, which combines the therapeutic actions of green synthesized CuO NPs and 5-Fu. The lack of cytotoxicity in cells treated with the blank gel also highlights its biocompatibility and suitability as a delivery system for anticancer agents.

#### Antibacterial assay

**Fig. 10:**
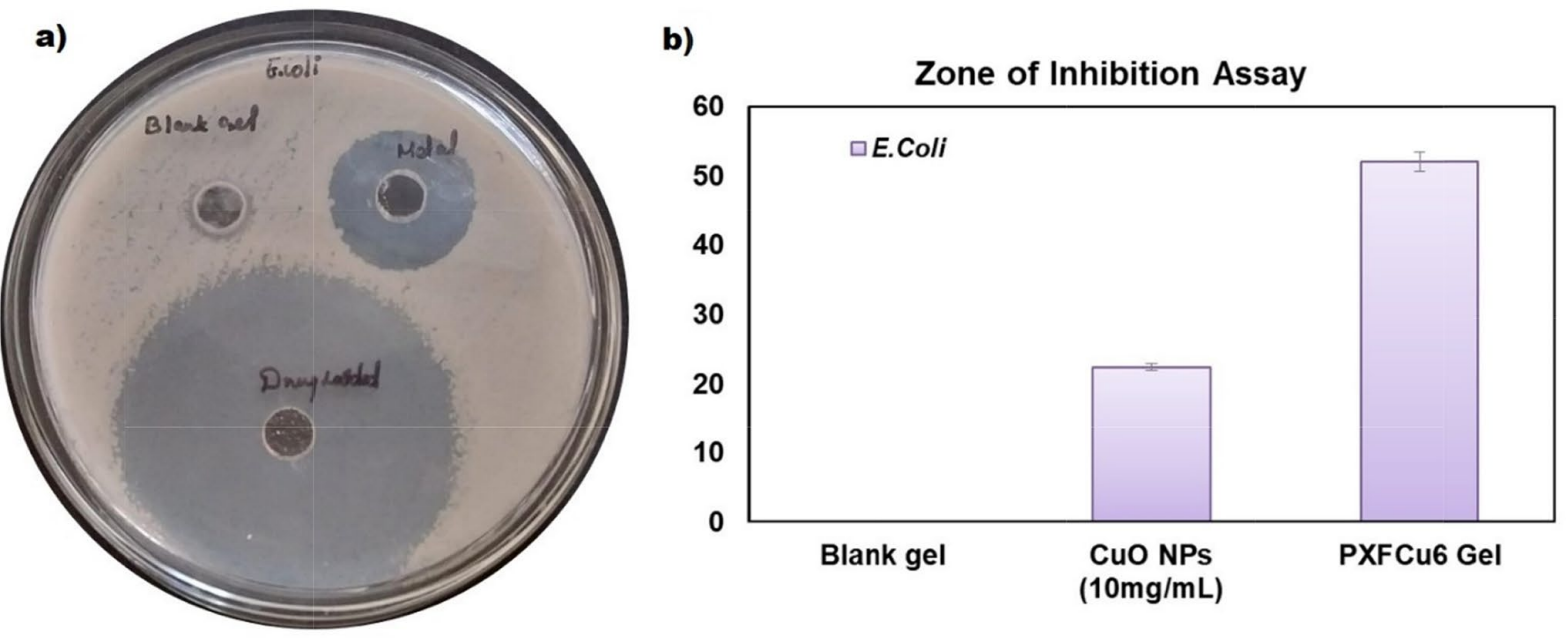
The Zone of inhibition assay on *E. coli* presented in the agar-wall diffusion plate (**a**) and the bar diagram represent the inhibition of the Zone of *E. coli* plate (**b**) [Data represent as mean ± SD (n = 3)]

The antibacterial zone of inhibition assay was performed to determine the antibacterial properties of the *T. dioica*-mediated CuO NPs and the *T. dioica*-mediated CuO NPs in combination with 5-Fu in a gel formulation (PXFCu6 gel) against *E. coli* growth and infections. The results indicated that the plain gel contained no specific inhibition zones, whereas the green synthesis CuO NPs exhibited substantial bacterial inhibition of 22.35 ± 49 mm. Similar to our findings, research studies have also proven the CuO NP's antibacterial activity, especially in *E. coli* inhibition [76, 77]. The PXFCu6 gel demonstrated the most extensive inhibition zones, 52.05 ± 1.37 mm on the bacterial plate, suggesting superior antibacterial activity against *E. coli* (Fig. 10). The synergistic effect of 5-Fu and CuO NPs in the gel formulation is responsible for the increased efficacy. CuO NPs have been recognized for their capacity to generate ROS, which induces cell death and disrupts bacterial membranes. Meanwhile, 5-Fu inhibits the synthesis of bacterial DNA, resulting in a more efficient elimination of bacteria [78, 79]. The gel formulation becomes even more effective in targeting and eliminating *E. coli* by integrating green synthesized CuO NPs and 5-Fu.

In the context of cervical cancer, which is often caused by severe HPV infection, the vaginal pH may frequently increase to levels above 4.5, making it less acidic [80]. This less acidic environment is more conducive to the growth of various pathogenic bacteria, leading to infections in the vagina and nearby urinary tract, such as aerobic vaginitis and urinary tract infections (UTIs). These infections are commonly caused by the gram-negative bacterium *E. coli*, responsible for 80–90% of UTIs [81, 82]. To understand the capacity to prevent the growth of *E. coli*, the PXFCu Gel was tested, and the result suggests that it has superior antibacterial activity against *E. coli*. Its synergistic antibacterial properties may help maintain a healthy vaginal flora by inhibiting the proliferation of *E. coli*. Additionally, the gel's sustained release of 5-Fu and green synthesized CuO NPs assures extended antibacterial activity, thereby further protection against bacterial proliferation. The PXFCu6 gel can decrease the probability of aerobic vaginitis and urinary tract infections (UTIs), which are frequent complications associated with altered vaginal pH and bacterial imbalances, by preventing the proliferation of *E. coli* [81, 83]. The gel's biocompatible properties guarantee its permanence, thereby enhancing vaginal health and providing continuous protection during cervical cancer treatment.

#### Stability assessment

The stability study of the PXFCu6 gel was conducted over a three-month period under two distinct storage conditions: 4 °C and 25 °C with 60% relative humidity (RH) (Table 4). The gel's viscosity, pH, drug release profile, phase separation, and microbial growth were systematically monitored. As detailed in Table 4, the PXFCu6 gel exhibited optimal stability when stored at 4 °C, maintaining consistent viscosity, pH, and drug release profile throughout the study. In contrast, at 25 °C, the gel demonstrated a decline in viscosity and an increase in pH over time, accompanied by a decrease in drug release rate. Notably, phase separation began after three months at this higher temperature. These changes are attributed to temperature-induced degradation and phase separation of the gel components. No microbial or fungal contamination was detected at either temperature despite these variations. These findings underscore the superior stability of PXFCu6 gel at 4 °C, making it the preferred condition for long-term storage to preserve the gel's integrity and therapeutic efficacy.

**Table 4:** Stability study of PXFCu6 anticancer gel

Duration	Stability condition	Viscosity (cP)	pH	Drug release (h)	Phase separation	Microbial growth
0 day 1st month	–	8426 ± 40	4.60 ± 0.18	≤ 8 h	No	No
	4 °C ± 0.5 °C (no RH)	8240 ± 59	4.6 ± 0.04	≤ 8 h	No	No
	25 °C ± 2 °C (60% RH ± 5% RH)	7841 ± 45	4.8 ± 0.07	≥ 7 h	No	No
3rd month	4 °C ± 0.5 °C (no RH)	8243 ± 78	4.6 ± 0.08	≤ 8 h	No	No
	25 °C ± 2 °C (60% RH ± 5% RH)	7188 ± 84	5.0 ± 0.11	7 h >	A slight cloudy phase was observed at the bottom	No

Data represented as mean ± S.D (n = 3).

## Conclusions

The study emphasizes developing and evaluating green synthesized CuO NPs and 5-Fu loaded gel for the synergistic effect on HeLa Cells. The experiments successfully synthesized *T. dioica* dried seeds extract-mediated CuO NPs with an average particle size of 26.44 ± 3.28 nm, displaying a nearly spherical shape as observed through HRTEM. XRD analysis confirmed the crystallinity of the CuO NPs, with a crystal size of 8.85 nm, which is beneficial for enhancing anticancer efficacy. The zeta potential was measured at −23.7 mV, indicating good colloidal stability in an aqueous medium. MTT assay results showed that the CuO NPs effectively inhibited the growth of HeLa cells, with previous studies suggesting that the cytotoxicity is primarily due to ROS generation, leading to DNA damage, membrane disruption, and, ultimately, apoptosis. FTIR analysis confirmed no undesirable interactions between the CuO NPs, 5-Fu, and other excipients in the PXFCu gel formulation. Additionally, the pH and viscosity measurements confirmed the suitability of the gel for local administration in the cervical region. HRSEM analysis revealed an even distribution of CuO NPs within the gel matrix. The gel-based delivery system facilitated a controlled and sustained release of the anticancer agents more than 8 h, potentially maintaining therapeutic drug concentrations at the targeted site for an extended period, thus enhancing its effectiveness for localized cervical cancer treatment.

The MTT assay evaluated the cytotoxicity of *T. dioica*-mediated CuO NPs on HeLa cells, showing an IC_50_ of 42.8 ± 0.24 μg/mL. In comparison, 5-Fu showed an IC_50_ of 19.3 ± 0.49 μg/mL, and PXFCu6 gel exhibited the most potent cytotoxicity with an IC_50_ of 11.82 ± 0.21 μg/mL. Morphological analysis confirmed apoptosis in treated cells, while the blank gel showed no cytotoxic effects, demonstrating its biocompatibility as a delivery system. PXFCu6 gel was 3.62 times more potent than CuO NPs alone and 1.63 times stronger than 5-Fu alone. The gel system has efficacy in triggering apoptosis in HeLa cells, with increased cytotoxic effects resulting from the combined action of green-produced CuO NPs and 5-Fu. The antibacterial zone of inhibition assay evaluated the efficacy of *T. dioica*-mediated CuO NPs and PXFCu6 gel against *E. coli*. While the plain gel showed no inhibition, the CuO NPs displayed significant inhibition (22.35 ± 49 mm), and the PXFCu6 gel exhibited the largest zone (52.05 ± 1.37 mm), demonstrating the superior antibacterial activity, indicating that it may protect the vagina and lower the risk of infections like aerobic vaginitis and UTIs. The stability study of PXFCu6 gel over three months at 4 °C and 25 °C showed optimal stability at 4 °C, maintaining viscosity, pH, and drug release. At 25 °C, a mild viscosity decreased, pH increased, and phase separation occurred, but no microbial contamination was found.

The simultaneous use of *T. dioica*-facilitated CuO NPs and 5-Fu in a gel formulation (PXFCu gel) presents a hopeful, promising approach for targeted drug administration. Local delivery systems can increase drug concentrations specifically at the tumor site, improving treatment effectiveness while reducing harmful effects on the rest of the body. The gel formulation has features and capabilities appropriate for direct administration to the cervical tumor location through intravaginal routes, probably using the applicator. Future research should concentrate on in vivo studies to further validate the gel's safety and efficacy. Furthermore, investigating the integration of alternative bioactive chemicals and refining the gel formulation might amplify its therapeutic capacity. Clinical studies are essential for assessing the gel's efficacy in real-life situations and determining the most appropriate dose and frequency of administration.

## Data Availability

The authors may provide raw data upon request.
